# Fusion color space analysis of tongue image characteristics in different stages of metastatic colorectal cancer and three-class model construction

**DOI:** 10.3389/fphys.2026.1926646

**Published:** 2026-09-03

**Authors:** Zhenyi Lin, Fan Chen, Zhenzheng Zhu, Dingxuan Wu, Weiwei Zhou, Shuning Ding, Leitao Sun, Guanjun Jiang

**Affiliations:** 1 The First Affiliated Hospital of Zhejiang Chinese Medical University (Zhejiang Provincial Hospital of Chinese Medicine), Hangzhou, China; 2 College of Computer Science and Technology, Zhejiang University of Water Resources and Electric Power, Hangzhou, China; 3 The School of Basic Medical Sciences of Zhejiang Chinese Medical University, Hangzhou, China; 4 Longyou Branch of Zhejiang Provincial Hospital of Chinese Medicine, Quzhou, China

**Keywords:** color space, colorectal cancer, deep learning, oligometastasis, tongue image

## Abstract

**Objective:**

Color spaces were used to analyze the color characteristics of tongue image in different stages of metastatic colorectal cancer (mCRC), and a three-class model was constructed to predict the different tongue characteristics during the mCRC progression.

**Methods:**

Tongue images of CRC patients were collected and classified into non-metastatic, oligometastatic, and extensive metastatic groups according to tumor metastasis. The color components in HSB, Lab, and YCrCb color spaces were measured from tongue images and the differences in color characteristics were quantitatively analyzed. Based on the modified YOLO and combined with the expert model and multilayer perceptron (MLP) fusion framework, the three classification models of tongue images were constructed.

**Results:**

Most of the quantitative results showed the quantitative results for oligometastasis were the smallest values among the three metastatic stages. The S* and b* values at the edge of the tongue and the b* values in the middle of the tongue showed the quantitative results for oligometastasis were the highest values in the three stages. Besides, Cr* values at the edge of the tongue and S* values in the middle of the tongue showed a decreasing trend. In addition, all expert models constructed in the study demonstrated stable convergence on the training corpus within 100 epochs. The overall accuracy of the fused MLP classifier for tongue characteristics classification was 83.3%.

**Conclusions:**

In this study, tongue feature variations across different stages of CRC were successfully analyzed. A well-performing three-classification tongue image analysis model based on the YOLO architecture was initially constructed.

## 1 Introduction

Colorectal cancer (CRC) is among the most prevalent malignant tumors affecting the digestive system globally, bring an increasingly serious public health challenge (Sung et al., 2021). Almost 150,000 new cases and more than 50,000 deaths from CRC are reported each year in the United States (Siegel et al., 2021). While the overall incidence of CRC has declined among older adults due to improved screening and lifestyle modifications, there is a growing trend of cases shifting toward younger individuals, particularly those under 50 (Keum and Giovannucci, 2019). CRC is frequently characterized by its high recurrence and metastasis rates, coupled with a low survival rate (Biller and Schrag, 2021). Recent advances in multidisciplinary management, including systemic therapy and localized interventions for primary and metastatic lesions, have significantly improved survival outcomes in metastatic colorectal cancer (mCRC) patients. Despite this progress, the 5-year relative survival rate for stage IV disease remained markedly low at 15.6%, in stark contrast to the 65.0% observed across all stages of CRC. (Centers for Disease Control and Prevention: Cancer Stat Facts: Colorectal Cancer SEER 18 2011-2017. 2022. https://seer.cancer.gov/statfacts/html/colorect.html). It is observed that the majority of patients either failed to exhibit an initial response or presented with symptoms only when the disease had already progressed to an aggressive stage (Brenner and Chen, 2018). Furthermore, 20-50% of cases initially diagnosed as localized disease will subsequently develop metastatic recurrence (Väyrynen et al., 2020; Ciardiello et al., 2022). A minority of metastatic patients qualify for curative resection, but a substantial proportion might develop oligometastatic disease amenable to local ablative therapies (Riihimäki et al., 2016).

Hellman and Weichselbaum first proposed the “oligometastasis” hypothesis, positing that metastatic progression represented a spectrum rather than invariably leading to widespread dissemination (Hellman and Weichselbaum, 1995; Weichselbaum and Hellman, 2011). This intermediate “oligometastatic” state was characterized by a limited number of slowly progressing metastases and favorable tumor-host biological interactions (Katipally et al., 2023). While traditional oncological paradigms considered metastatic disease exclusively suitable for systemic palliative therapy, emerging clinical evidence demonstrates that selected patients may derive significant benefit from aggressive local interventions, including surgical resection, radiotherapy, and various ablation techniques. Numerous clinical studies have established that CRC patients with isolated liver metastases can achieve long-term survival through combined surgical and systemic therapy (Morris et al., 2023). Comparable outcomes have been documented for other metastatic sites, particularly pulmonary metastases (Stewart et al., 2018; Osterlund et al., 2021). NCCN guidelines endorse local therapies (surgery, radiotherapy, or ablation) combined with systemic treatment (chemotherapy, targeted agents, or immunotherapy) for oligometastatic patients, offering the potential for prolonged disease control or even cure (Cervantes et al., 2023). Thus, accurately assessing the extent of metastasis in CRC patients as early as possible is crucial for implementing effective therapeutic strategies.

Traditional Chinese Medicine (TCM), with its patient-centered comprehensive treatment theory, has become the dominant discipline in personalized diagnosis and treatment. It takes holism and treatment based on syndrome differentiation as the basic principle (Li and Xu, 2011), and takes inspection, smell, inquiry and pulse-taking as the four basic methods of disease diagnosis (Zhang et al., 2021). Inspection includes inspection of the whole body, local conditions and tongue diagnosis, among which tongue diagnosis is an important content of inspection and the key for syndrome differentiation and treatment. Practice has proved that it can reflect the changes in the clinical condition during the progression of the disease. For instance, the morphological and color characteristics of the tongue sublingual veins (SLVs) were used to predict the clinical symptoms of menstruation in women (Tanaka, 2015), while macroglossia and purpura of the tongue and lips were regarded as a sign of myeloma-associated systemic amyloidosis (Rogers and Bruce, 2004). Moreover, with the development of deep learning technology, tongue diagnosis objectification has gradually become a method of disease screening and diagnosis. Shi et al (Shi et al., 2023). added objective tongue image data to baseline data to improve the efficacy of lung cancer prediction models. Li et al (Yuan et al., 2023). established a novel technology for gastric cancer diagnosis based on tongue image and coating using a deep learning model.

**Figure 1:**
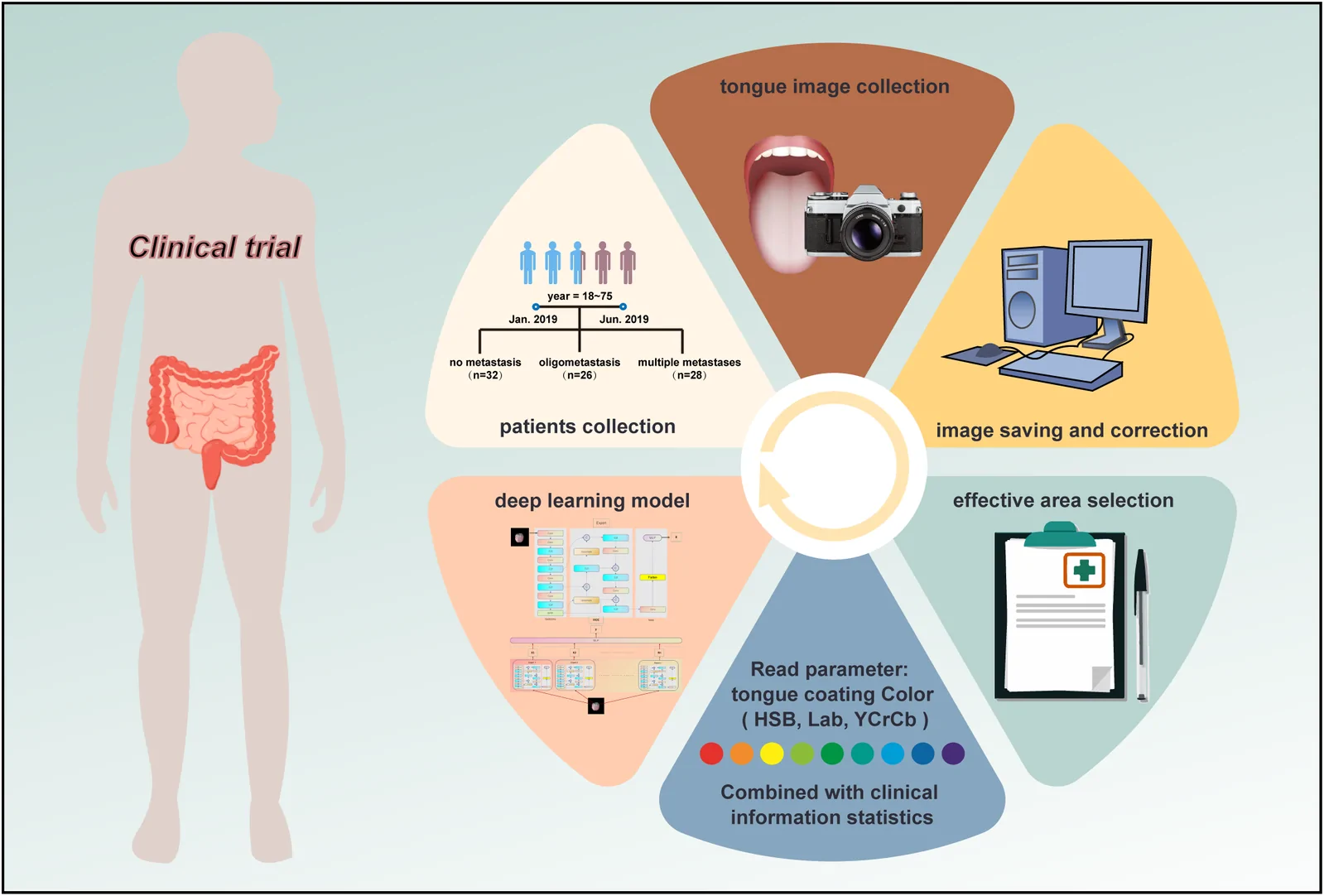
Graphical abstract.

Therefore, this study collected different tongue images of mCRC at different clinical stages, in order to explore the changes in tongue images during the progression of CRC by introducing the color space of HSB, Lab, and YCrCb. The three-class classification model was established employed the deep learning technology to learn and summarize the commonalities of tongue images among different stages of CRC, thus allowing for the prediction of different CRC stages (**Figure 1**). This study aims to explore the biological significance of tongue characteristics, and unleash the potential of tongue diagnosis as a screening and diagnostic method for CRC, and provide a unique technological path for the development of precision medicine.

## 2 Materials and methods

### 2.1 Clinical cases

This is a *post-hoc* ancillary study of a previously established prospective parent cohort. The parent study was a prospective observational cohort that pathologically confirmed colorectal cancer patients between February 2019 and June 2019 from Oncology Department of the Zhejiang Provincial Hospital of Chinese Medicine. For the present analysis, we performed a secondary data analysis using the previously unanalyzed tongue images collected at baseline from this cohort. Patients were included if they had available tongue images and complete clinical records at baseline. The Clinical Research Ethics Committee of the First Affiliated Hospital of Zhejiang Chinese Medical University approved the study protocol (2024-KLS-012-01). Although ethics approval was obtained after patient recruitment, this reflects the retrospective nature of the present ancillary analysis, not a deviation from ethical standards; all patients provided informed consent at the time of enrollment in the parent study.

#### 2.1.1 Diagnostic criteria

Oligometastase is defined by the presence of a limited number of metastases, and can achieve complete macroscopic clearance of all metastatic lesions through surgery, ablation, or other means, representing a potentially curable stage of metastasis (Guckenberger et al., 2020; Cremolini et al., 2026). In this study, oligometastase was defined as 1–3 metastatic lesions, with a total number of organs ≤2, and both the primary tumor and the metastatic lesions could be treated through surgery or ablation. Metastases below this threshold were categorized as non-metastasis, while those exceeding it were classified as extensive metastases.

According to the *Comprehensive Diagnosis and Treatment Guidelines for Colorectal Cancer* (2023 Edition), CRC patients are classified into four types of syndromes, including the spleen-kidney yang deficiency syndrome manifested as aversion to chills, the liver-kidney yin deficiency syndrome manifested as night sweats, the blood stasis syndrome with scaly and dry skin, and the damp-heat syndrome manifested as thick and sticky stools.

#### 2.1.2 Inclusion criteria

1. Patients pathologically diagnosed with CRC;
2. Participants aged 18 to 75;
3. Patients who voluntarily participate and sign the informed consent form;
4. Patients who can cooperate in completing basic information filling and tongue images collection.

#### 2.1.3 Exclusion criteria

1. Patients with obvious abnormalities in the oral environment (e.g., consumption of high-pigment foods on the same day);
2. Patients who are pregnant or lactating;
3. Patients with concurrent other malignancies;
4. Patients with severe internal medical diseases, including those of the heart, lung, liver, kidney, endocrine system, as well as autoimmune diseases, uncontrolled active infections, or mental illnesses.

### 2.2 Tongue images collection and correction

Tongue images were collected in a closed dark-light room with fixed, standardized artificial lighting. Each patient was instructed to enter the room in the early morning after waking, without having brushed their teeth, drunk water, eaten, or smoked. The patient was then seated in a fixed seat and asked to protrude the tongue. Tongue images were taken by a researcher using the same camera at a fixed angle. After the images were imported into a computer and corrected, a professional team selected the valid regions of the tongue images to establish an image database. Detailed procedures and the image collection steps are provided in the **Supplementary Material**.

### 2.3 Chromaticity analysis of tongue images

After the collection of tongue images, based on the human perception of color, we project the collected images into three color spaces: Lab, HSB, and YCrCb.

#### 2.3.1 HSB color space

HSB describes three basic characteristics of color, i.e. Hue (*H*), Saturation(*S*), and Brightness (*B*). *H* represents the color that is reflected or propagated through an object, *S* refers the intensity or purity of the color, indicating the proportion of gray components in the hue. *B* is the relative brightness of a color, which is usually measured by a percentage of black (0%) to white (100%).

#### 2.3.2 Lab color space

Lab is a sensory-homogeneous model, consisting of *L* and two chromatic components *a* and *b*. The *L* represents luminosity, and the higher the *L* value, the brighter it is. The *a* and *b* respectively represent the red-green axis and the yellow-blue axis. The positive value of a indicates red, otherwise, indicates green. Similarly, *+b* indicates yellow, and *-b* indicates blue.

#### 2.3.3 YCrCb color space

The principle of YCrCb formation is similar to the principle of human visual perception. YCrCb also consists of three components. The *Y* value represents luminosity, the *Cr* value represents the difference between the red signal and the luminance value, and the *Cb* value represents the difference between the blue signal and the luminance value.

The tongue images were opened with Adobe Photoshop 2020, and a few representative areas were selected as tongue color measurement points in each tongue image at the tip, edge, and middle of the tongue. Take care to exclude the tongue moss coverage and reflective points, with a sampling size of 3×3 mean pixels.

### 2.4 Constructing a three classifications model based on the improved YOLO architecture

Two physicians with intermediate or higher professional titles will annotate each tongue image without communication, extracting key diagnostic indicators (including tongue color, tongue coating thickness, coating color, dryness, tongue shape, overall deficiency-excess degree, etc.). If there are differences in the results, the third senior professional title physician will annotate again, and the final annotated result will prevail. After excluding samples with poor image quality or incomplete labeling, a multi label dataset is constructed for deep learning analysis, enabling the model to predict different CRC transitions based on tongue images.

All experiments were conducted on an NVIDIA A6000 GPU, with models implemented in PyTorch.

#### 2.4.1 Model design

This study adopts a two-stage analytical framework:

##### 2.4.1.1 Indicator regression stage

**Figure 2:**
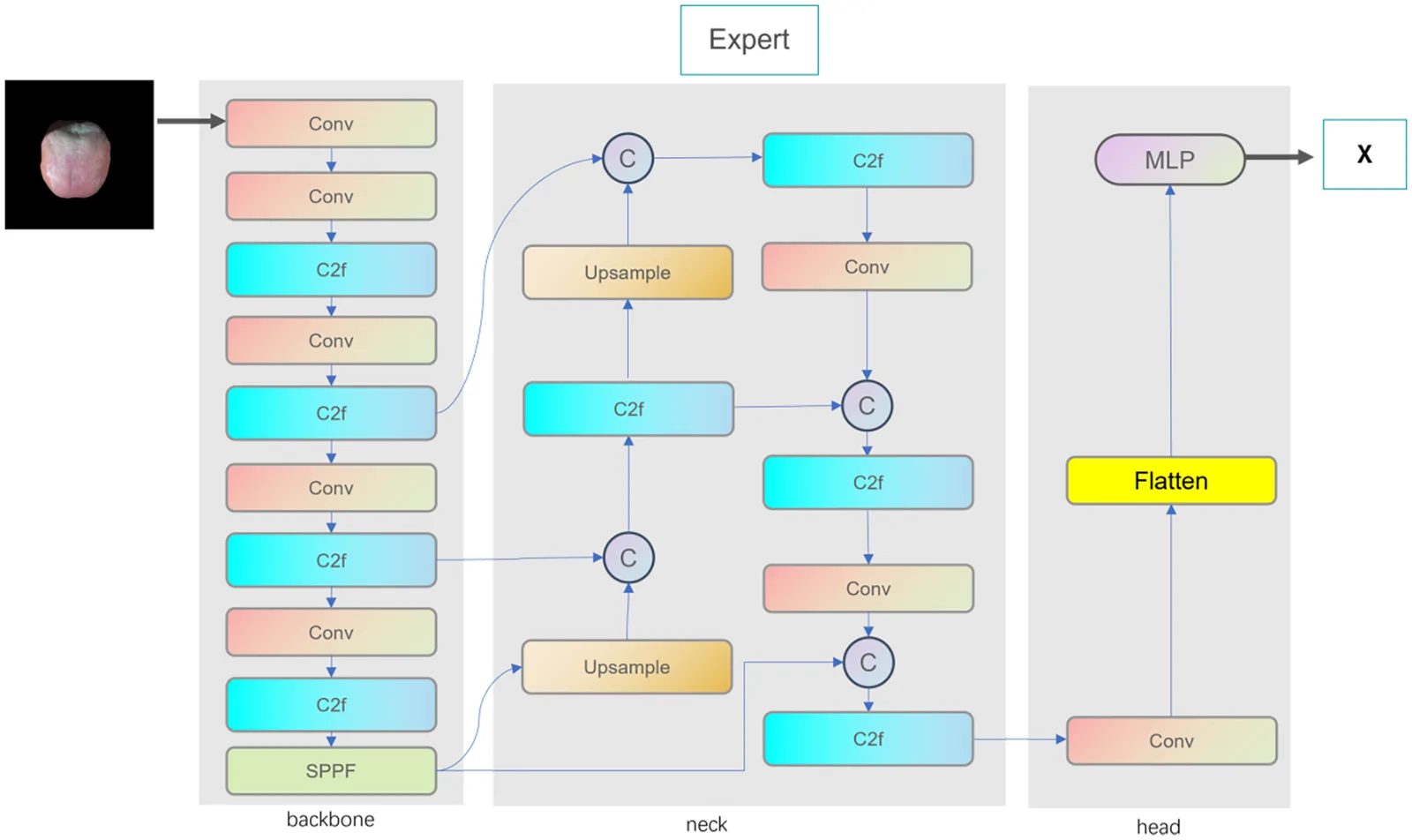
Schematic diagram of improved YOLO structure.

A set of specialized “Expert Models,” based on an enhanced YOLO architecture, was developed, with each model dedicated to predicting a specific indicator. Instead of conventional bounding box or categorical probability outputs, the detection head of each model was modified to generate continuous floating-point predictions via a Flatten + Multilayer Perceptron (MLP) structure (**Figure 2**).

##### 2.4.1.2 Fusion classification stage

**Figure 3:**
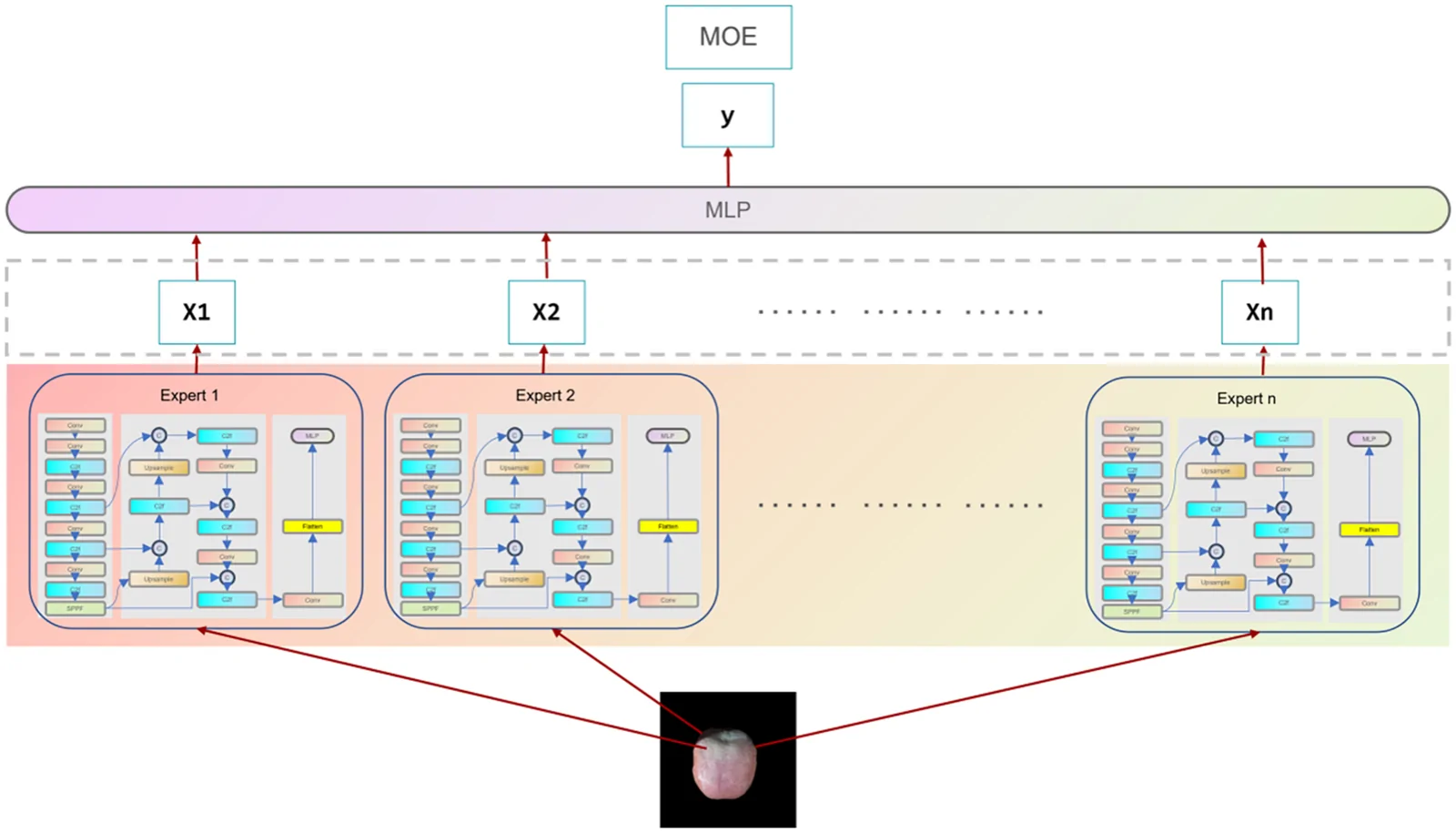
MoE model framework diagram.

The outputs from all expert models were concatenated into a comprehensive feature vector and fed into a fusion classifier. The classifier, structured as an MLP, comprised two hidden layers (ReLU activation) and a Softmax output layer, enabling multi-class prediction of tongue characteristics (**Figure 3**).

#### 2.4.2 Model training

The expert models were trained on 80% of the dataset, with the remaining 20% reserved for validation. Input images were resized to 224×224 pixels (RGB). Training spanned 100 epochs using the Adam optimizer (initial learning rate: 1×10^-4^). Performance was assessed using mean squared error (MSE), mean absolute error (MAE), root mean squared error (RMSE), precision, and recall. The performance metrics of the expert models (MSE, MAE, and RMSE) were calculated on the validation set.

#### 2.4.3 Model advantages

Inspired by the Mixture of Experts (MoE) paradigm, this modular design allows: Independent training and optimization of each expert model, minimizing inter-task interference. Scalability for future integration of additional diagnostic indicators.

#### 2.4.4 Classification model performance evaluation

Firstly, the three classes were defined according to the clinical grouping in Section 2.1: class 0 = non-metastatic, class 1 = oligometastatic, and class 2 = extensive metastatic.

The three-class classification performance of the fusion model was evaluated on a validation set of 18 samples (class 0: 7 cases; class 1: 5 cases; class 2: 6 cases). The model correctly classified 15 cases and misclassified 3 cases, achieving an overall accuracy of 83.33% (Wilson 95% confidence interval: 60.78%–94.16%). Standard multiclass balanced accuracy was 82.22%, Macro-F1 was 81.82%, and weighted F1 was 83.33%. Multiclass Matthews correlation coefficient (MCC) and Cohen’s κ were 0.752 and 0.749, respectively, indicating good agreement between predicted and true labels.

Class-specific metrics were as follows:

Class 0: all 7 samples correctly classified; sensitivity, specificity, precision, F1-score, class-balanced accuracy, and negative predictive value all = 100.00%.

Class 1: 4 samples correctly classified, 1 misclassified as class 2; sensitivity 80.00%, specificity 84.62%, precision 66.67%, negative predictive value 91.67%, F1-score 72.73%, class-balanced accuracy 82.31%, false-positive rate 15.38%, false-negative rate 20.00%, Jaccard index 57.14%, one-vs-rest MCC 0.614.

Class 2: 4 samples correctly classified, 2 misclassified as class 1; sensitivity 66.67%, specificity 91.67%, precision 80.00%, negative predictive value 84.62%, F1-score 72.73%, class-balanced accuracy 79.17%, false-positive rate 8.33%, false-negative rate 33.33%, Jaccard index 57.14%, one-vs-rest MCC 0.614.

The relatively lower sensitivity of class 2 indicates that under-detection of this class constitutes one of the main error sources.

Overall summary metrics: macro-averaged precision and recall both 82.22%, macro-averaged specificity 92.09%, Macro-F1 81.82%, macro-averaged Jaccard index 71.43%; weighted-average precision, recall, F1-score and Jaccard index 84.07%, 83.33%, 83.33% and 73.81%, respectively; micro-averaged precision, recall and F1-score all 83.33%, micro-averaged Jaccard index 71.43%. The geometric mean of the per-class recalls was 81.10%.

Error-distribution analysis showed that all three misclassifications occurred exclusively between classes 1 and 2 (one class-1 sample predicted as class 2; two class-2 samples predicted as class 1); no confusion involving class 0 was observed. The model predicted 6 samples as class 1 and 5 samples as class 2, whereas the true counts were 5 and 6, respectively, indicating that the dominant error pattern is the misassignment of class-2 samples to class 1.

### 2.5 Statistical analysis

SPSS 27.0 and Excel 2021 were used to analyzed data. Continuous variables with non-normal distributions were presented as median (IQR), with intergroup differences assessed by the Kruskal-Wallis H test. Categorical baseline variables were reported as frequencies and percentages, and compared across groups using the Fisher-Freeman-Halton exact test with 10 000 Monte Carlo simulations, especially for small expected counts. For pairwise comparisons, Student’s t-test was employed for normally distributed data with equal variances, Welch’s t-test for unequal variances, and the Wilcoxon rank-sum test for non-normal data. Statistical significance was set at a two-sided *P*-value < 0.05.

## 3 Results

### 3.1 Baseline characteristics

According to the inclusion and exclusion criteria, a total of 86 CRC patients’ clinical information and tongue images were collected, and the above population was categorized according to the definition of oligometastasis: 32 patients had no metastasis, about 37%; 26 patients were defined as oligometastasis, about 30%; and 28 patients had extensive metastasis, about 33%. Baseline demographic and clinical features of the three groups are summarized in **Table 1**. No statistically significant inter-group differences were observed in age, tumor location, T-stage, N-stage, surgical intervention, radiotherapy, chemotherapy, and TCM treatment (*P* > 0.05). The overall baseline profiles were well-balanced among groups.

**Table 1:** Baseline demographic and clinical features.

Characteristic	Total (N = 86)	no metastasis (N = 32)	Oligometastasis (N = 26)	Extensive metastasis (N = 28)	*P* value
**Age, years, Median (P25-P75)**	60.00 (53.00-67.00)	60.00 (52.75-65.00)	62.00 (57.75-68.50)	58.50 (51.25-66.50)	**0.290**
Sex, n (%)					0.561
Male	52 (60.47%)	17 (53.13%)	17 (65.38%)	18 (64.29%)	
Female	34 (39.53%)	15 (46.87%)	9 (34.62%)	10 (35.71%)	
Tumor location, n (%)					0.566
Colon	44 (51.16%)	19 (59.38%)	11 (42.31%)	14 (50.00%)	
Rectum	36 (41.86%)	10 (31.25%)	13 (50.00%)	13 (46.43%)	
Colorectal	6 (6.98%)	3 (9.37%)	2 (7.69%)	1 (3.57%)	
T-stage, n (%)					0.194
T1 or T2	10 (11.63%)	5 (15.63%)	4 (15.39%)	1 (3.57%)	
T3 or T4	46 (53.49%)	20 (62.50%)	12 (46.15%)	14 (50.00%)	
Tx	30 (34.88%)	7 (21.87%)	10 (38.46%)	13 (46.43%)	
N-stage, n (%)					0.571
N0	20 (23.26%)	10 (31.25%)	4 (15.38%)	6 (21.43%)	
N1 or N2	35 (40.70%)	13 (40.63)	13 (50.00%)	9 (32.14%)	
Nx	31 (36.04%)	9 (28.12)	9 (34.62%)	13 (46.43%)	
Surgery, n (%)					0.263
Yes	75 (87.21%)	30 (93.75%)	23 (88.46%)	22 (78.57%)	
No	11 (12.79%)	2 (6.25%)	3 (11.54%)	6 (21.43%)	
Radiotherapy, n (%)					0.352
Yes	11 (12.79%)	2 (6.25%)	4 (15.38%)	5 (17.86%)	
No	75 (87.21%)	30 (93.75%)	22 (84.62%)	23 (82.14%)	
Chemotherapy, n (%)					1.000
Yes	76 (88.37%)	28 (87.50%)	23 (88.46%)	25 (89.29%)	
No	10 (11.63%)	4 (12.50%)	3 (11.54%)	3 (10.71%)	
TCM treatment, n (%)					0.507
Yes	69 (80.23%)	27 (84.38%)	19 (73.08%)	23 (82.14%)	
No	17 (19.77%)	5 (15.62%)	7 (26.92%)	5 (17.86%)	

TNM staging was based on postoperative pathological findings where available; clinical staging was adopted for non-surgical patients.

Age was reported as median (P25‑P75) because the variable violated normal distribution assumption; categorical variables were presented as n (%).

The bold values represent the main descriptive statistics of baseline characteristics for the included patients. For categorical variables, bold numbers denote the case number and proportion for each subgroup; for the continuous variable age, the bold value corresponds to the median and interquartile range (P25‑P75).

**Figure 4:**
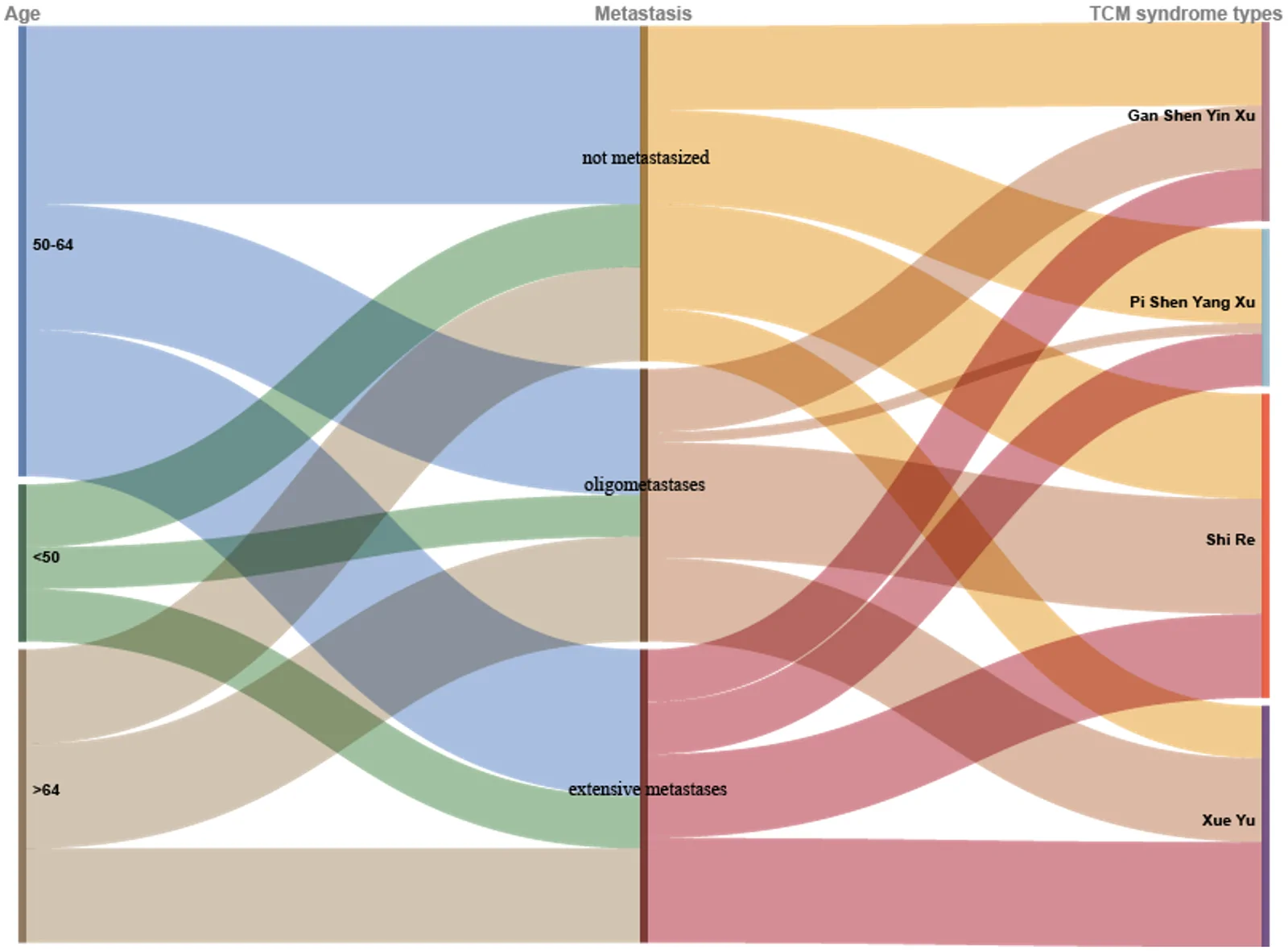
Metastasis status and TCM syndrome types across age groups.

The prevalence of Traditional Chinese Medicine (TCM) syndrome types in descending order is as follows: damp-heat syndrome (33.72%), blood stasis syndrome (26.74%), liver-kidney yin deficiency syndrome (22.09%), and spleen-kidney yang deficiency syndrome (17.44%) (**Figure 4**).

### 3.2 Quantitative analysis of tongue image parameters based on multiple color spaces

#### 3.2.1 Quantitative analysis of tongue image parameters in different stages of CRC

As shown in **Table 2**, it was found that as CRC progressed, most of the quantitative results showed a parabolic-like curve with an upward opening, which meant that the quantitative results for oligometastasis were always the smallest values among the three metastatic stages. The S* and b* values at the edge of the tongue as well as the b* values in the middle of the tongue showed a parabolic curve with a downward opening, which meant that the quantitative results for oligometastasis were the highest values in the three stages. Besides, Cr* values at the edge of the tongue and S* values in the middle of the tongue showed a decreasing trend.

**Table 2:** Quantitative analysis of tongue image parameters in different stages of CRC.

Color space	Parameters	No metastasis	Oligometastasis	Extensive metastasis
		33	26	28
The tip of the tongue	H	261.34 ± 151.28	215.65 ± 170.45	234.39 ± 160.93
	S	37.94 ± 9.4	33.15 ± 10.17	33.79 ± 12.37
	B	70.09 ± 12.52	64.5 ± 14.18	70.46 ± 12.18
	L	55 ± 9.63	53.12 ± 11.95	57.07 ± 10.11
	a	27.91 ± 8.57	22.04 ± 7.98	24.43 ± 9.27
	b	7.19 ± 7.22	7.08 ± 7.04	7.46 ± 9.13
	Y	129.85 ± 26.08	125.58 ± 27.25	135.45 ± 28.56
	Cr	160.94 ± 11.85	154.52 ± 9.95	156.61 ± 11.54
	Cb	122.11 ± 9	120.56 ± 5.72	121.05 ± 7.83
The edge of the tongue	H	263.22 ± 150.7	205.62 ± 173.07	222.75 ± 161.84
	S	30.97 ± 9.96	31.38 ± 10.32	30.57 ± 11.87
	B	72.81 ± 12.62	67.62 ± 12.53	73.36 ± 12.27
	L	60.16 ± 10.23	56.12 ± 10.47	60.93 ± 10.33
	a	22.84 ± 8.09	20.77 ± 8.45	22.39 ± 9.95
	b	6.88 ± 7.1	8.46 ± 6.83	6.36 ± 8.7
	Y	140.66 ± 25.43	134.69 ± 26.54	143.68 ± 32.89
	Cr	154.15 ± 9.57	153.8 ± 9.87	151.69 ± 13.37
	Cb	121.19 ± 7.57	119.73 ± 5.47	122.15 ± 9.79
The middle of the tongue	H	211.66 ± 156.83	129.15 ± 156.52	218.39 ± 154.32
	S	26.38 ± 10.79	25.46 ± 11.89	24.64 ± 13.01
	B	70.81 ± 15.58	64.96 ± 15.59	71.25 ± 13.44
	L	62.13 ± 14.05	57.54 ± 14.11	62.57 ± 11.71
	a	16.13 ± 10.39	13.69 ± 9.34	16.18 ± 9.63
	b	5.59 ± 9.24	6.92 ± 9.04	5 ± 10.28
	Y	144.85 ± 29.43	138.64 ± 35.45	147.22 ± 29
	Cr	148.35 ± 11.18	145.46 ± 11.14	147 ± 12.71
	Cb	121.86 ± 8.28	120.83 ± 8.36	122.54 ± 10.05

#### 3.2.2 Quantitative analysis of tongue image parameters in different TCM syndromes of CRC

As shown in **Figure 4**, significant differences were observed in the distribution of CRC stages across distinct TCM syndrome groups. The spleen-kidney yang deficiency syndrome group was dominated by “non-metastatic” cases (61.54%), while the liver-kidney yin deficiency syndrome group was dominated by “non-metastatic” cases (46.67%). The damp-heat syndrome group was mainly composed of “non-metastatic” (41.18%) and “extensive metastasis” (35.29%) cases, and the blood stasis syndrome group was dominated by “extensive metastasis” cases (60.00%).

The B* value was the largest in the liver-kidney yin deficiency syndrome group and the smallest in the blood stasis syndrome group (p<0.05). L* and Y* values were highest in the damp-heat syndrome group and lowest in the blood stasis syndrome group (p<0.05). In the middle part of the tongue, the a* value was the largest in the liver-kidney yin deficiency syndrome group and the smallest in the damp-heat syndrome group (p<0.05). No statistically significant differences were observed in HSB, Lab, and YCrCb color space measurements at the tongue tip across the four groups (**Table 3**).

**Table 3:** Quantitative analysis of tongue image parameters in different TCM syndromes of CRC.

Color space	Parameters	Spleen-kidney yang deficiency syndrome	Liver-kidney yin deficiency syndrome	Damp-heat syndrome	Blood stasis syndrome
		15	19	29	23
The tip of the tongue	H	279.93 ± 142.18	274.16 ± 143.27	206.34 ± 171.97	223.52 ± 165.16
	S	34.87 ± 9.46	38.05 ± 9.7	33.76 ± 10.06	34.65 ± 13.25
	B	72.2 ± 9.67	73.47 ± 11.08	68.62 ± 11.85	61.91 ± 15.62
	L	58.2 ± 7.96	57.42 ± 8.44	56.21 ± 10.87	49.78 ± 11.69
	a	25.93 ± 6.87	29.11 ± 8.82	23.14 ± 7.69	23.35 ± 10.63
	b	7.33 ± 7.15	7.05 ± 8.8	7.9 ± 7.09	6.52 ± 8.45
	Y	141.26 ± 21.28	131.2 ± 20.55	132.8 ± 28.68	119.56 ± 31.16
	Cr	159.83 ± 9.37	162.31 ± 11.11	155.6 ± 9.78	154.75 ± 13.7
	Cb	122.04 ± 10.69	120.66 ± 6.51	120.19 ± 6.35	122.73 ± 8.05
The edge of the tongue	H	209.73 ± 170.63	258.05 ± 155.25	230.24 ± 165.39	229.57 ± 162.9
	S	28.2 ± 12.13	33.89 ± 9.45	31.03 ± 10.51	30.26 ± 10.66
	B	75.93 ± 9.29	75.74 ± 11.32	71.41 ± 12.52	64.91 ± 13.38
	L	64.4 ± 7.86	61 ± 9.37	59.24 ± 11.33	54.22 ± 9.89
	a	20.4 ± 8.6	25.89 ± 8.05	21.41 ± 8.42	20.83 ± 9.53
	b	7.6 ± 8.63	8 ± 7.88	7.79 ± 7.24	5.48 ± 7.12
	Y	155.12 ± 23.74	137.15 ± 25.6	145.02 ± 31.47	125.56 ± 22.82
	Cr	151.05 ± 10.44	157.4 ± 8.85	153.2 ± 11.77	151.29 ± 11.48
	Cb	122.04 ± 8.35	119.77 ± 6.68	119.83 ± 7.94	123.04 ± 8.16
The middle of the tongue	H	223.93 ± 158.17	239.79 ± 156.88	119.66 ± 141.82	211.35 ± 161.94
	S	22.4 ± 12.29	32.26 ± 11.13	23.28 ± 11.66	24.87 ± 10.52
	B	72.2 ± 15.16	71.89 ± 12.6	70.83 ± 14.56	62.91 ± 16.34
	L	64.27 ± 13.2	59.42 ± 12.71	64.69 ± 12.93	55.09 ± 13.13
	a	15.2 ± 8.22	22.26 ± 10.8	10.55 ± 8.24	16 ± 8.56
	b	3.53 ± 8.89	5.89 ± 9.92	8.66 ± 9.45	3.61 ± 9.02
	Y	149.52 ± 25.42	136.06 ± 27.53	154.63 ± 32.32	132.6 ± 31.75
	Cr	147.33 ± 11.79	152.26 ± 12.89	143.46 ± 10.81	147.04 ± 10.36
	Cb	123.65 ± 7.93	121.27 ± 9.04	119.57 ± 9.17	123.73 ± 8.65

### 3.3 Three-classification model assessment

**Figure 5:**
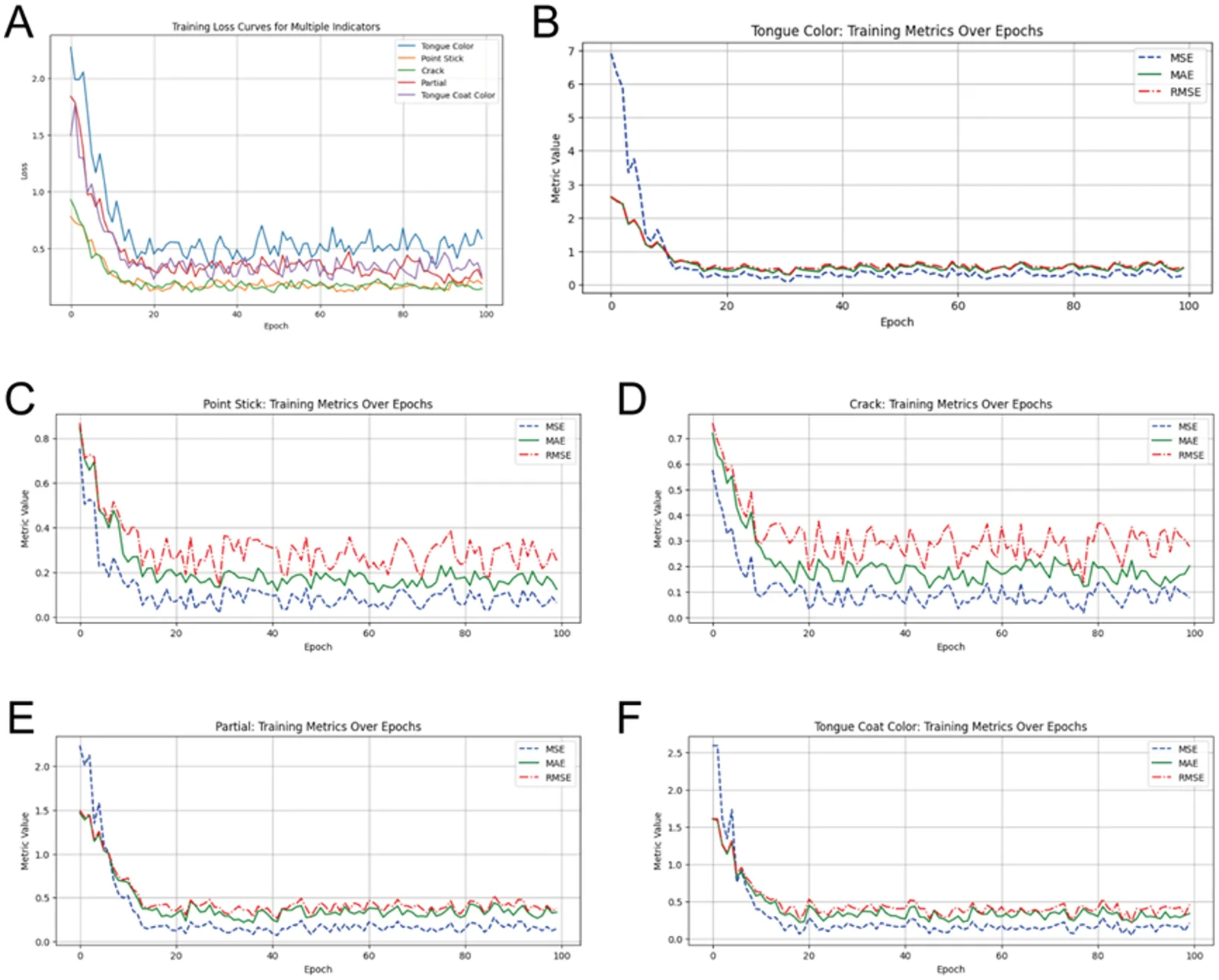
Tongue indicator chart. **(A)** Comparison of losses in each indicator; **(B–F)** MSE/MAE/RMSE for each indicator.

**Figure 6:**
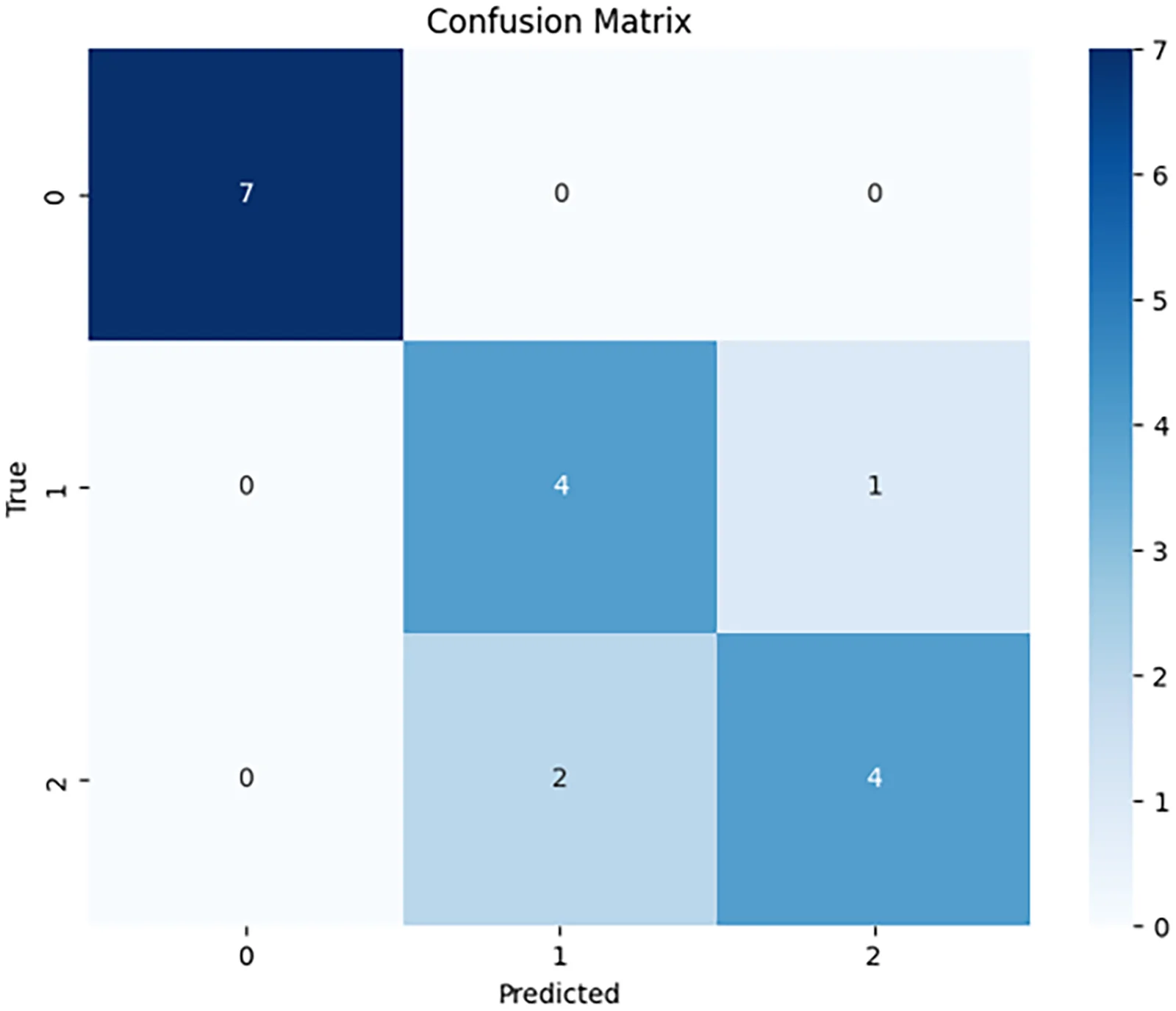
Confusion matrix for identifying CRC metastasis via tongue image. Metastatic status was coded as 0 for no metastasis, 1 for oligometastasis, and 2 for extensive metastasis.

All expert models constructed in the study demonstrated stable convergence on the training corpus as shown in **Figure 5**. The prediction performance of the tongue color model on the validation corpus was quantified using MSE, MAE, and RMSE, and the detailed results are shown in **Table 4**. Class-activation mapping (CAM) visualization showed that the model mainly focused on the mid-tongue and root regions, which are also frequently emphasized in TCM tongue diagnosis. The overall accuracy of the fused MLP classifier for tongue characteristics classification was 83.3%, with high precision and recall in distinguishing different metastasis types and predicting CRC subtypes (**Table 5**). However, the confusion matrix (**Figure 6**) showed that occasional misclassification occurred between the “oligometastasis” and “pan-metastasis” categories, which may be due to the overlapping metrics represented in these categories. The expert model combined with MLP fusion framework proposed in this paper has shown strong performance in automatic tongue image analysis, achieving effective indicator extraction and disease classification. The proposed framework demonstrates promising performance in automated tongue image analysis and may offer a feasible technical approach for the intelligent interpretation of TCM tongue diagnosis, with potential for further clinical investigation.

**Table 4:** Expert model indicators.

Indicator	Avg MSE	Avg MAE	Avg RMSE
Tongue Color	0.5820	0.6336	0.7629
Point Stick	0.0596	0.2090	0.2442
Crack	0.0553	0.2095	0.2352
Partial	0.2346	0.4144	0.4844
Tongue Coat Color	0.2291	0.4048	0.4786

**Table 5:** Precision and recall for different classifications.

	No metastasis	Oligometastasis	Extensive metastasis
Precision	1	0.67	0.8
Recall	1	0.8	0.67

## 4 Discussion

CRC is a highly prevalent gastrointestinal malignancy for which screening strategies need to be optimized, posing a significant public health challenge. Current screening modalities, including fecal tests, radiologic imaging, and endoscopy, are limited by their reliance on specialized equipment, invasive nature, and suboptimal cost-effectiveness (Ladabaum et al., 2020; Jain et al., 2022). Alarmingly, approximately 56% of patients are diagnosed at advanced stages (36% with locally advanced stage III and 20% with distant metastatic stage IV), leading to poor prognosis and substantial socio-economic burden (László, 2010), which underscores the critical demand for enhanced early screening systems.

Currently, the definition of oligometastatic disease is heterogeneous, with varying criteria such as ≤ 3 or ≤ 5 lesions. According to the ESMO functional definition, oligometastasis is characterized by: 1–5 metastatic lesions (with the possibility of >5 lesions if all can be completely eradicated); involvement of at most two organs; and controlled primary tumor (either previously resected or amenable to treatment) (Cremolini et al., 2026), several expert consensus statements for specific tumor types still adopted a threshold of 1–3 lesions (Ost et al., 2018). Moreover, evidence indicated that patients with 1–3 lesions achieve better treatment outcomes and prognosis than those >3 lesions (Okamoto et al., 2024). Therefore, to enhance the representativeness of our study results, and given that the patients in this study were enrolled relatively early (in 2019), it was decided that the 1–3 metastatic lesion group would continue to be used as the group for oligometastasis in this study.

Oligometastatic disease was represented the only potentially curable phase of metastatic cancer currently (Van Cutsem et al., 2016). Localized therapies [e.g., surgical resection, radiofrequency ablation (RFA), and stereotactic ablative radiotherapy (SABR)] can extend median survival to 40 months in oligometastatic CRC patients, contrasting sharply with the 20-month survival observed in extensively metastatic cases (Moretto et al., 2020; Rim et al., 2022; Yoo et al., 2023). Although advanced imaging techniques such as ¹^8^F-FDG PET/CT have demonstrated diagnostic utility for oligometastases (Samim et al., 2017), their reliance on invasive contrast administration necessitates the development of simpler, non-invasive diagnostic alternatives.

Tongue diagnosis is an important part of TCM diagnostics and has been systematically proven to be a non-invasive diagnostic method. Tongue images can reflect microbial changes in the oral cavity through quantifiable changes in the thickness of the tongue moss, moistening, and color of the tongue texture (Han et al., 2014; Cui et al., 2022). As the second largest microbial reservoir after the gut (Kitamoto et al., 2020), oral microbial dysbiosis is specific in the development of CRC: healthy controls exhibit significantly higher relative abundances of Neisseria (23.8%), Haemophilus (18.4%), and Porphyromonas (12.1%) compared to CRC patients (*p* < 0.01) (Han et al., 2016). This ecological imbalance is directly correlate with the tongue image features - thick yellow coatings correspond to 3.2-fold colonization of Clostridium difficile (Han et al., 2016), a known CRC-promoting pathogen. Furthermore, Wong et al (Wong and Yu, 2023). conducted a multicenter cohort study and mechanistically demonstrated that oral microbiota can regulate CRC through the “oral-gut axis”. Flemer et al (Flemer et al., 2018)’s research also clinically indicated that oral microbes can distinguish CRC patients from healthy individuals, confirming the potential of tongue-based microbiome as a non-invasive screening marker for CRC. In addition, Deng et al (Deng et al., 2025). found specific oral microbiota differences in patients with metabolic dysfunction-associated steatotic liver disease (MASLD) in different TCM patterns, allowing for specific tongue image characteristics in patients with different TCM patterns. The disease specificity of tongue image microorganisms is also evident in gastritis and gastric cancer. Studies have revealed differences in the microbial communities of tongue coating between gastritis patients and healthy individuals (Cui et al., 2019), demonstrating the potential for monitoring gastritis through tongue diagnosis and providing biological evidence for TCM tongue diagnosis. Research by Chen et al (Chen et al., 2024). indicates that gastric cancer patients exhibit downregulated expression of tongue coating proteins with temporal stability, resulting in tongue image characteristics attributable to tongue proteins that distinguish them from non-patients.

In this study, the tongue images of patients with CRC in different stages were analyzed via color spaces. It was found that S* and b* at the edge of the tongue and b* in the middle of the tongue were the largest values in the oligometastatic group, suggesting that the tongue edge of the oligometastatic patients had a greater saturation of color, and that the color of the tongue image at the edge of the tongue and in the middle of the tongue was yellowish. With the progression of CRC, Cr* at the tongue edge and S* in the middle of the tongue decreased, denoting that the color of the tongue edge gradually became lighter from red and the color of the middle of the tongue gradually became greyish in the process of CRC progression. Overall, most of the quantitative results in the oligometastatic group were the smallest, demonstrating a transition of tongue color from bright to grayish and back to bright during CRC progression. Thus, the study had found that quantitative parameters of the tongue image color space differ across various stages of CRC, which provides preliminary evidence supporting the potential feasibility of staging CRC based on tongue characteristics.

Meanwhile, the study found that patients with spleen-kidney yang deficiency syndrome and liver-kidney yin deficiency syndrome were predominantly non-metastatic, while patients with blood stasis syndrome mostly showed extensive metastasis. Analysis of tongue image color space parameters with different TCM syndromes has revealed that the a* value in the middle of the tongue was the highest in the liver-kidney yin deficiency syndrome group, indicating that the tongue body of patients with this syndrome was the reddest, consistent with the characteristic of “red tongue with scanty coating” in liver-kidney yin deficiency syndrome in TCM theory. At the same time, the B* value at the edge of the tongue in this group was the largest, but the L* and Y* values, which represent brightness, did not reach the maximum. It might be because B* represents relative brightness based on the maximum value of the RGB channel and the tongue body of patients with liver-kidney yin deficiency syndrome was reddish, which means the R channel value is high, thus pushing up the B* value, while L* and Y* are less affected by redness. Patients with damp-heat syndrome had a moister tongue, so the L* and Y* values at the edge of the tongue in this group were the largest. In addition, the middle of the tongue in these patients was mostly covered with relatively thick and greasy fur, which means the red color is lighter, hence the smallest a* value in the middle of the tongue. In the blood stasis syndrome group, the three indices representing brightness (B*, L*, Y*) at the edge of the tongue were all the smallest, indicating that the tongue body was duller in this group, consistent with the characteristic of “dark tongue” in blood stasis syndrome in TCM theory.

In this study, color spaces analysis was performed on the tip, edge, and middle portions of the tongue respectively, which is in line with the principle of TCM tongue diagnosis. The color space parameters digitized the subjective judgment of TCM tongue diagnosis and contributed to the objectification. Meanwhile, color space is a digital way to describe the color pattern of human visual perception, which can more comprehensively describe color perception than the RGB space. In addition, this study introduced three different color spaces for analysis, which utilized multiple color characteristics of the tongue image and may have enhanced the overall analysis efficacy. However, color spaces only make use of the tongue color, while TCM tongue diagnosis is not only judged from this, but also involves other aspects such as the thickness of the tongue moss and the shape of the tongue, etc. To refine tongue diagnosis and enhance the objectivity and precision of tongue characteristics assessment, the study introduced deep learning techniques to construct a tri-class classification model for CRC based on tongue image analysis.

The model was based on a modified YOLO architecture that combined an expert model and a MLP fusion framework. It successfully analyzed multiple metrics such as tongue color, fissures, and tongue papillae, and had significant potential to improve the objectivity and accuracy of tongue image analysis. Through class activation mapping, the model achieved an accuracy of 83.3% for metastasis classification and its focus on diagnostically critical regions (e.g., middle and root of the tongue) was observed, which is consistent with TCM principles and aligns with clinical needs, supporting the biological plausibility of the model. Notably, the framework demonstrated robust performance in quantifying tongue characteristics, as evidenced by low error metrics on key metrics: exceptionally high accuracy was obtained for the cleft and point stick features (MSE < 0.06, MAE < 0.21), while the tongue color analysis maintained a clinically acceptable error margin (MSE: 0.58, RMSE: 0.76). The classification reliability of the model was further confirmed by its balanced precision values (up to 1.0) and recall values (up to 0.8) for non-metastatic cases, effectively reducing false positives and false negatives, a key requirement for cancer screening tools. As highlighted in a recent study on the standardization of AI-driven medical imaging (Zhu et al., 2023), these results offer better consistency than certain conventional methods. And the model offers a novel approach toward digitizing TCM diagnostics by translating subjective visual assessments into repeatable metrics (e.g., crack quantification with an RMSE of 0.24). In addition, the improved YOLO architecture reduces hardware requirements (25% less memory usage) while maintaining accuracy through channel compression (28% fewer parameters) and hybrid attention mechanisms (CBAM + coordinated attention), making it more suitable for resource-limited clinical environments.

A degree of confusion (20% crossover error) was identified between the oligometastatic and poly-metastatic categories, which may primarily reflect biological realities rather than solely algorithmic weaknesses, and reflects the diagnostic challenges that clinicians face in distinguishing between migratory tumor stages.

However, as an initial proof-of-concept study, the study has some limitations. 1.The sample size is relatively small, and external validation is lacking. Although tongue images were acquired under standardized conditions with manual ROI selection by blinded TCM practitioners, factors such as smoking, oral inflammation, nutritional status, and anticancer treatments may influence tongue appearance and were not fully controlled in this small-sample exploratory study. 2.Tongue characteristics may be influenced by ethnicity, diet, and geographic region; a single-center cohort cannot fully capture such variability. 3.Compared with current state-of-the-art AI approaches, our model relies on a relatively conventional algorithm with limited flexibility. 4.The model uses only tongue characteristics and does not incorporate multimodal data such as laboratory tests or imaging findings. 5. Although this study documented metastatic profiles among CRC patients with different TCM patterns, the intrinsic mechanistic links between TCM syndromes and CRC metastasis remain poorly understood. In the future, we should establish large-scale, multicenter cohort studies, with systematic documentation of potential confounders, collect and integrate multi-omics information, and improve multidimensional correlation assessments to enhance predictive accuracy. Longitudinal follow-up is also needed to verify whether the model’s predictions align with actual patient prognosis. Standardized tongue image acquisition devices should be introduced to improve imaging stability. Additionally, subsequent research may dissect associations between TCM syndromes and CRC metastasis from the oral-microbiota perspective, which could yield novel auxiliary biomarkers for CRC metastasis screening.

## 5 Conclusion

In this study, tongue characteristics variations across different stages of CRC were successfully analyzed through the application of HSB, Lab and YCrCb color spaces, which realized the objectification of TCM tongue diagnosis. The correlation between tongue characteristics and CRC at different metastatic stages was identified, suggesting that tongue image analysis may provide a potential approach for objective CRC stage assessment. In addition, this study combined traditional TCM tongue diagnosis with cutting-edge deep learning technology and a three-classified tongue image analysis model based on the YOLO architecture was constructed. Although it was a preliminary effort, the model demonstrated robust performance, effectively translating subjective tongue diagnostic experience into quantifiable image features, for instance achieving a low MSE of 0.58 in quantifying tongue color, which may contribute to the standardization of TCM diagnostics and provide a quantitative framework for objective tongue image analysis.

## Data Availability

The original contributions presented in the study are included in the article/**Supplementary Material**. Further inquiries can be directed to the corresponding authors.
