# Supplementary material for: Fusion color space analysis of tongue image characteristics in different stages of metastatic colorectal cancer and three-class model construction
Source: Front Physiol. 2026 Sep 3;17:1926646. doi: 10.3389/fphys.2026.1926646 (PMC13581709; doi:10.3389/fphys.2026.1926646)
Supplement: Supplementary file 1 [file DataSheet1.docx]

**Supplementary material**

The process of obtaining tongue images

1. Patient preparation: Participants were instructed not to brush their teeth or rinse their mouths before image acquisition to minimize the transient effects of dietary intake and oral cleaning procedures on tongue characteristics.

2. Time, environment and equipment preparation:

Tongue images were collected at a fixed time in the early morning under fasting conditions. Patients were seated in a fixed seat within a closed dark‑light environment and standardized artificial light. The seat can be adjusted up and down to ensure that patients of different heights have the same height of the mouth during the photo-taking process. The researcher used a Canon77D digital camera with effective pixels of 24.2 million, an ISO sensitivity of 1600, an aperture value of f/8, the angle of shooting is 10° with the horizontal line into the angle of prismatic.

3. Tongue image collection process:

Each participant extended their tongue naturally out of the mouth for full exposure of the tongue surface, keeping the tongue spread flat and the tongue tip slightly downward. The patient holds an X-rite Color Checker Classic Mini AISI 24-color standard mini-color card and places it next to the tongue as a reference aid. The image format is saved in the format of RAW + JPEG. If the time of tongue extension is exceeded or the image is unsatisfactory, it’s necessary to repeat the photography.

4. Image correction:

Use Adobe manufacturer's Lightroom Classic to open the captured tongue and 24-color card images, and create the International Color Consortium characteristics (ICC Profile) required for color correction.Then import the tongue images into the Lightroom software, and correct the tongue images with the created characteristics file as the standard.

5. Effective area selection:

Two researchers licensed physicians of traditional Chinese medicine with rich clinical experience independently selected the representative region of each tongue image. Their results were comprehensively compared and integrated to generate the final unified region for subsequent analysis. In cases of substantial disagreement regarding the selected representative regions between the two physicians, a third senior licensed TCM physician with extensive clinical experience was invited for reassessment to reach a consensus. Additionally, an expert panel of three professors specializing in medical oncology in TCM was established. In cases of disagreement among the researchers regarding the selected regions, the panel discussed the issue and provided the most professional opinion.
